# Schema Therapy for Emotional Dysregulation: Theoretical Implication and Clinical Applications

**DOI:** 10.3389/fpsyg.2016.01987

**Published:** 2016-12-22

**Authors:** Harold Dadomo, Alessandro Grecucci, Irene Giardini, Erika Ugolini, Alessandro Carmelita, Marta Panzeri

**Affiliations:** ^1^Department of Neuroscience, University of ParmaParma, Italy; ^2^Parma Schema Therapy CenterParma, Italy; ^3^Clinical and Affective Neuroscience Lab, Department of Psychology and Cognitive Sciences, University of TrentoRovereto, Italy; ^4^Bologna Schema Therapy Center BolognaBologna, Italy; ^5^Firenze Schema Therapy Center FirenzeFirenze, Italy; ^6^Italian Society for Schema TherapySassari, Italy; ^7^Department of Developmental Psychology and Socialisation, Padua UniversityPadova, Italy

**Keywords:** emotion regulation, schema therapy, experiential techniques, personality disorder, psychotherapy, cognitive behavioral therapy

## Abstract

The term emotional dysregulation refers to an impaired ability to regulate unwanted emotional states. Scientific evidence supports the idea that emotional dysregulation underlies several psychological disorders as, for example: personality disorders, bipolar disorder type II, interpersonal trauma, anxiety disorders, mood disorders and post-traumatic stress disorder. Emotional dysregulation may derive from early interpersonal traumas in childhood. These early traumatic events create a persistent sensitization of the central nervous system in relation to early life stressing events. For this reason, some authors suggest a common endophenotypical origin across psychopathologies. In the last 20 years, cognitive behavioral therapy has increasingly adopted an interactive-ontogenetic view to explain the development of disorders associated to emotional dysregulation. Unfortunately, standard Cognitive Behavior Therapy (CBT) methods are not useful in treating emotional dysregulation. A CBT-derived new approach called Schema Therapy (ST), that integrates theory and techniques from psychodynamic and emotion focused therapy, holds the promise to fill this gap in cognitive literature. In this model, psychopathology is viewed as the interaction between the innate temperament of the child and the early experiences of deprivation or frustration of the subject’s basic needs. This deprivation may lead to develop early maladaptive schemas (EMS), and maladaptive Modes. In the present paper we point out that EMSs and Modes are associated with either dysregulated emotions or with dysregulatory strategies that produce and maintain problematic emotional responses. Thanks to a special focus on the therapeutic relationship and emotion focused-experiential techniques, this approach successfully treats severe emotional dysregulation. In this paper, we make several comparisons between the main ideas of ST and the science of emotion regulation, and we present how to conceptualize pathological phenomena in terms of failed regulation and some of the ST strategies and techniques to foster successful regulation in patients.

## Background and Theory

The term “Emotional regulation” refers to a series of strategies aimed at modulating and adjusting unpleasant emotional experiences (John and Gross, 2004; Gross, 2011). Emotional regulation is a multidimensional construct composed of the following traits: (1) awareness and acceptance of emotions; (2) skills to engage in behaviors aimed at a target; (3) flexible use of appropriate strategies to modulate the context’s intensity and the duration of the emotional response (Pedersen et al., 2014). Deficits in these areas are considered indicative of emotional dysregulation and are an indicator of psychopathology (John and Gross, 2004). Adopting effective strategies of emotional regulation is considered one of the fundamental aspects of individual adaptation. In fact, different scientific evidence demonstrated that emotional dysregulation is one of the main important factors in different disorders as, for example: cluster b personality disorder, bipolar disorder, interpersonal trauma, anxiety disorder, mood disorder, and post-traumatic stress disorder. Schema Therapy (ST) is a relatively new treatment approach to treat chronic Axis I and Axis II disorders (Young et al., 2003). According to this model, stable and enduring Early Maladaptive Schemas (EMSs) are at the core of chronic Axis I and Axis II disorders (Young et al., 2003). The term “schema” is derived from the theory of information processing, which maintains that the information is sorted in human memory by theme (Williams et al., 1997; Vonk, 1999). The idea is that the experiences are stored in our autobiographical memory by means of diagrams from the early years of life (Zajonc, 1980, 1984; Conway and Pleydell-Pearce, 2000). The patterns consist of sensory perceptions, experience, emotions, and the meaning attributed to them, so that early childhood experiences are stored at a non-verbal level (Freeman, 1981; Greenberg and Safran, 1989; Christianson and Engelberg, 1999; Young, 2005; Rijkeboer and Huntjens, 2007). Schemas act as filters through which individuals order, interpret and predict the world. EMSs have been shown to mediate the relationship between adverse childhood experiences and adult psychopathology (Carr and Francis, 2010). Because EMSs are considered ego-syntonic, therapists believe that clients with chronic difficulties lack the motivation to change them. Young incorporated a range of technique from Gestalt and Emotion-Focused Therapies (Perls and Baumgardner, 1975; Safran et al., 1988; Greenberg and Safran, 1989), particularly imagery work and empty chair dialogs (Kellogg, 2004) for treating and changing EMSs. Recent insights have lead to the view that complex Personality Disorders (PD) are not characterized by one set of pathogenic EMSs, but by different sets of EMSs activated by the same trigger, and having the same purpose, that can be activated as a group of schemas. In therapy, dealing with many schemas at the same time can result very difficult. For this reason, Young introduced the concept of Schema Modes in 2002. Schema Modes (from here, we will simply name them Modes), are relatively independently organized patterns of thinking, feeling and behaving that underlie the different states of consciousness; they can be directly observable and measurable, because they represent the moment-to-moment emotional and cognitive states and coping responses that are active at a given point in time. Modes are triggered by emotional events and an individual may shift from one mode into another very rapidly (oscillating dyads in psychodynamic terms). Modes were introduced to ST in order to explain the abrupt changes in thoughts, feelings and behaviors displayed by patients with severe PD (Young, 2002). In this way, the mode concept describes the rapid shifting in emotion and behavior demonstrated by patients suffering from severe PD (Young et al., 2003; Lobbestael et al., 2007). Compared to standard CBT, ST assigns a central role assigned to the concept of reparative therapeutic relationship (e.g., limited reparenting) and emotion-focused experiential techniques (e.g., imagery rescripting and chair work). These relational and experiential techniques can overcome some of the limitations of the standard CBT approach such as the poor attention given to elaborate and problematic emotional states. Improved cognition does not necessary mean improved emotion regulation (Grecucci and Job, 2015; Grecucci et al., 2015a, 2016). Greenberg and Safran (1984) provided evidence that rational cognitive language-based systems are independent from emotion based systems. To understand this, the model of Interacting Cognitive Subsystems was proposed (ICS; Teasdale, 1993; Waltz and Rapee, 2003), which distinguishes between two systems: the propositional coding system of meanings – that is based on language, which can be assessed and directly influenced by sensory information – and the implicational coding system of meanings – that elaborates experiences from a wide variety of sources, including specific patterns of indirect sensory input -. It follows that, if a therapist wants to change dysfunctional behavioral patterns, he/she has to work on the level of this implicational coding system and activate target emotional states. Following other psychotherapeutic approaches (Bowlby, 1969; Singer, 1974; Samuels and Samuels, 1975; Pope and Singer, 1978; Singer and Pope, 1978, 1980; Shorr, 1983; Sheikh, 1984; Burke et al., 1992; Frankel, 1993; Guntrip, 1995; Field and Horowitz, 1998; Fonagy, 1998), ST implements several emotion focused techniques rather than simple cognitive techniques, to foster emotion regulation. There is now empirical evidence that imagery work can have more impact than rationalist methods in fostering emotional change (see for example, Holmes et al., 2007). Another key factor in ST is the role of the therapeutic relationship. ST focuses on painful childhood experiences that were central to the development of the patient’s EMSs. Thus, ST involves the endeavor to re-experience and communicate the most vulnerable states of childhood, those in which the child desperately needed the care of adults but was not getting it (Young et al., 2003). The aim of this paper is to summarize theoretical implications of this model, empirical evidence and clinical application of ST in the management of emotional dysregulation, and to build bridges with the science of emotion regulation. We believe ST holds the promise to provide means to modulate severe dysregulated emotions as shown by PDs.

## Schema Therapy Model of Emotion Dysregulation and Emotion Regulation

In the last decades, emotion regulation has been increasingly considered as a focal point to address psychological disorders. In ST emotions and emotion regulation are strictly linked to the concept of schema mode. This concept is the essential and most complex aspect of the theoretical model proposed by Young et al. (2007; Lobbestael et al., 2005, 2008, 2009). A mode is an intense predominant dysregulated emotional state linked to a pattern of thinking, feeling and behaving based on a set of specific frustrated needs. Usually the modes are activated by external stimuli or internal states, are transient by definition and may comprise both adaptive and maladaptive responses (Young et al., 2007; Lobbestael et al., 2010). In socio-cognitive terms, the modes are the conception of the self that are active at a given time. They are the part of the self, or the identity of that person, that leads the way in which the subject him/herself anticipates, sees, and responds to the world around him/her (Kellogg and Young, 2006). In psychodynamic terms, a mode can resemble the concept of the object relation dyad active in the interpersonal situation (Clarkin et al., 2007). In particular, a dysfunctional mode is characterized by maladaptive schemas or coping responses erupting into distressing emotions, avoidance responses or self-defeating behaviors that influence an individual’s behavior and control his/her emotional functioning. The mode theory’s basic concept is that different mental states have different purposes and are related to different basic needs. The therapist’s first goal is to understand and conceptualize the subject’s model of functioning. This is done to simplify the work with the patient without being simplistic, helping him/her to understand his/her way of functioning. In the next paragraphs we show how every Mode is associated with either (1) Dysregulated emotions, or with (2) Dysregulatory strategies.

There are four Mode macro-categories (Young, 2002). The first macro-category of modes is the maladaptive *Child Modes* that developed when certain basic emotional needs were not adequately met in childhood. In terms of the science of emotion regulation, *Child Modes* are characterized by specific dysregulated emotions (anger, shame, sadness, etc.). With dysregulated emotions we indicate an exaggerated aspect of on of the components of the emotional response (onset, duration, strength, type or expression). The second macro-category of modes is the dysfunctional *Coping Modes* that reflect dysfunctional regulatory strategies or coping styles (overcompensation, avoidance or surrender). In terms of the science of emotion regulation, Coping modes are problematic regulatory strategies that may produce a momentary relief on the short run (for example, avoiding a situation that triggers the emotions associated with the EMS), but cause and maintain dysregulated emotional states on the long run (lack of interpersonal intimacy and attachment). The third macro-category of modes is the dysfunctional *Parent* modes that reflect internalized attitudes and opinions of the parents (or other significant persons or even social and peer groups) toward the patient as a child. Parent modes are the primary source of dysregulated emotions. In terms of emotion regulation science, these Modes are dysregulatory mechanisms that generate the most severe dysregulated emotions (for example, a Punitive parent Mode that induces self-hate and contempt toward the self). The last macro-category of modes is the integrative adaptive modes, that encompasses the *Healthy Adult* mode, which includes functional cognitions, thoughts and behaviors (Arntz et al., 2012), and the *Happy Child*, which feels at peace because all core emotional needs are currently met (Simeone-DiFrancesco et al., 2015). In terms of the science of emotion regulation, Happy Adult may be viewed as a collection of self-soothing, positive reappraisal like-, and acceptance based- regulatory strategies that regulate emotions and produce a Happy Child state of mind.

The first macro-category, concerning the Child modes, includes different emotional states. It includes three categories (Arntz et al., 2012).

The first category of Child Modes is named *Vulnerable Child* mode. It encompasses most EMSs and most of the suffering felt by patients. From this mode many modes that belong to the other two categories of child modes can derive, as well as dysfunctional coping modes (Arntz and Jacob, 2012). Exaggerated emotions of sadness, anguish, and shame characterize the mode of this category.

• *Lonely Child*. In this mode the patient feels emotionally empty, lonely and socially unacceptable, not worthy of being loved. Dysregulated sadness characterizes this mode.
• *Abandoned and Abused Child*. In this mode the patient feels sad, scared, alone, unworthy and unlovable: he/she feels the enormous pain and fear of abandonment caused by his/her abusive history, which expresses itself through depressive, fearful, desperate, and inferiority feelings. This mode can be evoked by perceptions of threatened abandonment and abuse. Severe anguish characterize this mode.
• *Humiliated and Inferior Child*. In this mode the subject feels incapable of managing responsibilities. The person in this mode shows strong regressive tendencies, he/she wants to be taken over. Usually we observe this mode in people who have developed poor autonomy and poor self-sufficiency. Dysregulated shame characterizes this mode.

Dysregulated anger, with different levels of expression, characterizes the second category of Child modes:

• *Angry Child.* This mode is characterized by feelings of anger, frustration and impatience because the patient’s needs have neither been considered nor satisfied. He/she may rebel against this alleged grievance, making pretentious or flawed demands, but does not attack others.
• *Stubborn Child.* This mode is a subtype of the *Angry Child*. Individuals feel angry but do not show anger openly. Instead, they persist passively, so stubborn in their positions or requests that they are deemed unreasonable by others.
• *Enraged Child*. In this mode the subject experiences stronger levels of anger that lead to uncontrolled aggression like hurting people or damaging objects. Aggression is out of control, and its goal is to destroy or eliminate the alleged assailant. The patient shows affectivity similar to that of a furious and uncontrollable child.

Dysregulated impulsivity characterize the third category of Childs modes:

• *Impulsive Child.* This mode refers to a person in which all locked emotions discharge impulsively, immediately and directly in order to meet his/her needs or desires, without being able neither to postpone their gratification nor to predict the consequences of his/her actions.
• *Undisciplined Child*. This mode describes an extremely frustrated person, unable to make efforts in order to fulfill routine or boring tasks, who, consequently, easily decides to give up.

See **Table 1** for a summary.

**Table 1:** Categories of dysregulated emotions in relation to modes and therapeutic strategies: child modes.

Modes category (subcategory)	Dysregulated emotion	Dysfunctional regulation strategy	Effects	Therapeutic strategy
Vulnerable Child (Lonely Child, Abandoned and Abused Child, Humiliated and Inferior Child)	Exaggerated sadness Anxiety	Self blame	Vulnerability Fragility Deprivation Exclusion	Imagery rescripting Reparenting in and extra-session Cognitive or behavioral techniques Limited reparenting
Angry Child (Angry Child, Stubborn Child, Enraged Child)	Exaggerated anger	Blame others	Impulsivity Interpersonal problems	Venting anger Limiting destructive expressions of anger or rage Increase ability to tolerate frustration Limited reparenting Cognitive techniques (e.g., using a diary to identify mode triggering situations) Behavioral techniques (e.g., role playing present situations etc.)
Impulsive Child Undisciplined Child	Emotions displayed with no control	Attack Interrupt Blame others Ignore others	Impulsivity Frustration Spoiled behavior Impatience Lack of control	Increase ability to find a realistic way to meet hedonistic needs Increase ability to tolerate frustration Therapeutic relationship


The second macro-category focuses on maladaptive coping modes. Parallels can be made with the concepts of defense mechanisms in psychodynamic terms, and with dysfunctional regulatory strategies (Gross, 2011; Grecucci et al., 2013; Grecucci and Job, 2015). It includes three categories.

The first category of dysfunctional Coping modes concerns the Avoidance strategy:

• *Detached Protector.* This mode is characterized by emotional and psychological withdrawal of the individual, who suppresses his/her feelings, depersonalizes him/herself and does not feel linked to or in contact with others. Therefore, feelings of emptiness, boredom and abulia are typical in this context.
• *Detached Self-soother*. This mode refers to an emotionally detached person, who tries to suppress and silence his/her emotions by compulsively and excessively committing to distracting and soothing activities, such as eating, watching TV, abusing drugs and having promiscuous sex.
• *Angry Protector.* In this mode the patient usually covers what he/she is really feeling with a stream of resentment and anger. They use a ‘wall of anger’ to protect themselves from others who are perceived as threatening. Displays of anger serve to keep others at a safe distance to avoid being hurt.
• *Avoidant Protector.* In this mode the patient usually avoids triggering by behavioral avoidance; he/she keeps away from situations or cues that trigger distress. The difference between *Detached Protector* and *Avoidant Protector* is that the former tends to inhibit or decrease the feeling of emotions, whereas the latter is characterized by interpersonal and situational avoidance.

In terms of emotion regulation science, these coping strategies belong to the class of “distancing” strategies, and produce an excessive down-regulation of (positive and negative) emotions (Grecucci et al., 2013, 2015a).

See **Table 2** for a summary.

**Table 2:** Categories of dysregulated emotions in relation to modes and therapeutic strategies: dysfunctional coping modes.

Modes category (subcategory)	Dysregulated emotion	Dysfunctional regulation strategy	Effects	Therapeutic strategy
Compliant Surrender	Reduced anger Assertiveness	Passivity Self defeating	Abuse acceptance Submission Masochism	Chair work to bypass and overcome avoidance coping mode Validation and empathic confrontation Identification and reappraisal of the mode through cognitive and experiential techniques
Detached Protector (Detached Protector, Detached Self-soother, Angry Protector, Avoidant Protector)	Down regulation of every emotion	Interpersonal detachment Isolation of affect Passive aggressive stance	Detachment Not caring Withdrawal Emptiness Depersonalization Self soothing behaviors	Chair work to bypass and overcome avoidance coping mode Validation and empathic confrontation Identification and reappraisal of the mode through cognitive and experiential techniques
Over-compensator (Self-Aggrandizer, Bully/Attack, Attention Seeker, Over-Controller, Manipulator, Predator)	Exaggerated grandiosity Anger Sense of dominance	Devaluing others Attack others	Arrogance Control Dominance Manipulation Exploitation Attention seeking	Chair work to bypass and overcome overcompensator coping mode Validation and empathic confrontation Identification and reappraisal of the mode through cognitive and experiential techniques Limit placing

The second category of dysfunctional Coping modes, diametrically opposed to the Avoidance coping strategies, is the Overcompensation that is composed of six modes:

• *Self-Aggrandizer.* In this mode the patient acts egoistically, shows little empathy for the needs and feelings of others and thinks he/she should not follow the rules like others do. He/she behaves competitively, hatefully, abusively. The subject is quite self-absorbed, craving the admiration of others, and showing superiority. Usually the emotion associated with this mode is anger, activated when someone threatens his/her status.
• *Bully/Attack.* This mode is characterized by the will to strategically harm others physically, psychologically, verbally and through antisocial or criminal actions. The emotion that characterizes this mode is often anger and the feeling of pleasure experienced when others are harmed.
• *Attention Seeker.* In this mode the patient attempts to get the attention and approval of others, with extravagant, inappropriate or exaggerated behaviors. Usually he/she tries to compensate for feelings of sadness and loneliness.
• *Over*-*Controller.* In this mode the patient tries to protect him/her-self from danger, real or perceived, by focusing on external details and brooding. There are two distinct subtypes: the *Perfectionist Over-Controller*, focused on perfectionism to gain control and prevent critical or misfortunes, the *Paranoid Over-Controller*, which is suspicious and focuses on supervision and is concerned by the malicious intent of others’ controlling behavior. Both these modes usually face the demanding parents’ attempts to make the child feel incompetent and not good enough.
• *Manipulator*. This mode manipulates, lies and frauds to obtain a specific goal that is usually to damage others or to avoid punishment
• *Predator*. This mode is focused on the elimination of a threat, a rival, an obstacle, in a merciless, cold and calculating way. Unlike the bully attack mode that is a hot mode of expressing anger, the predator instead is very cold and ruthless.

In terms of emotion regulation science, these coping strategies may be seen as variations of reappraisal strategies (Gross, 2011), as the individual reinterpret himself in an excessively positive way and interpret others in a devaluing way. This causes excessive emotions of power, dominance attributed to the self, as well as excessive negative emotions toward others (e.g., disgust, rage etc.).

The third category of dysfunctional Coping modes is the Surrender strategy:

• *Compliant Surrender.* This mode refers to a passive, servile, submissive behavior of someone constantly looking for everyone’s approval. Fearing conflict or refusal, the individual could even tolerate abuse and silence his/her needs or desires.

In terms of emotion regulation science, this coping strategy causes excessive fear of abandonment; often it causes also rage, that in this mode can be expressed only in a passive way.

See **Table 3** for a summary.

**Table 3:** Categories of dysregulated emotions in relation to modes and therapeutic strategies: dysfunctional parent modes.

Modes category	Dysregulated emotion	Dysfunctional regulation strategy	Effects	Therapeutic strategy
Punitive Parent	Exaggerated guilt Shame Contempt Disgust	Self attack Self devaluation Self punishment Self blame	Self directed abuse	Chair work to deal and overcome punitive parent mode Imagery rescripting to become aware of emotional needs and help the patient modify the situation in order to adequately meet needs Helping to express emotions and needs using healthy ways to deal with emotions Identification and reappraisal of the mode through cognitive and experiential techniques Active confrontation by the therapist to deal and overcome punitive parent mode using limited reparenting
Demanding or Critical Parent	Exaggerated sense of responsibility Guilt		Striving for high status Self neglect Humility Efficiency Rigidity Work addiction Lack of spontaneity Lack of pleasant activities	Chair work to deal and overcome punitive parent mode Imagery rescripting to become aware of emotional needs and help the patient modify the situation in order to adequately meet needs Helping to express emotions and needs using healthy ways to deal with emotions Identification and reappraisal of the mode through cognitive and experiential techniques Active confrontation by the therapist to deal and overcome punitive parent mode using limited reparenting

The third macro-category of modes includes the figures concerning the Dysfunctional *Parent*: the *Punitive Parent* and the *Demanding Parent*. These modes usually derive from parents or other attachment figures (Young et al., 2003). Nevertheless, they can derive also from internalized social or religious authority, peers, etc. (Simeone-DiFrancesco et al., 2015). They intrude as negative automatic thoughts (Beck and Emery, 1985) and can be theorized as toxic parental introjects (Freud, 1917), that patients hear as “voices inside the head.”

• *Punitive Parent*. This mode represents the interiorized voice of very critical and punitive attachment figures. This mode makes the patient afraid he/she did something wrong, sees him/herself as evil and worthless because of his/her feelings and desires. As a consequence, self-directed anger and hate develop and the patient punishes him/herself in one or another way.
• *Demanding Parent*. This mode represents the interiorized voice of very demanding and impossible to please attachment figures. This mode makes patients constantly feels under pressure, for he/she aims at reaching excessively high standards and goals. This mode constantly tells to you that you have to be perfect in order to be accepted by others. Moreover, others’ needs are almost always considered as more important and overriding than their own. While Demanding Parent makes one feel always wrong, Perfectionist Over-controller makes one feel loved and accepted.

These Parent modes are in our view the source of primary emotion dysregulation in the patient, and may be seen as a class of self-attacking/self-blaming strategies (in psychodynamic terms) that creates unbearable negative affects inside the patient.

The last macro-category of mode encompasses *Healthy Adult* and *Happy Child* modes:

• *Healthy Adult*. This mode presents significant adaptive and mediation functions between the different identified elements. It harbors and embraces the *Vulnerable Child*’s vulnerability; sets strict limits and boundaries on the *Angry* and the *Impulsive Child*’s behaviors; encourages and supports the *Happy Child*’s functionality; fights to replace the maladaptive coping strategies and, finally, neutralizes or limits the influence of his/her dysfunctional parents. Moreover, this mode also accomplishes appropriate adult functions, such as working, adopting care-giving behaviors and taking responsibilities. Furthermore, it engages in pleasant and stimulating adult activities, such as sex, cultural and esthetic interests and sports.
• *Happy Child.* This mode allows a person to feel loved, accepted, understood, safe and spontaneous because his/her core needs are been fulfilled.

When the patient is in these modalities, no dysregulatory strategies, nor dysregulated emotions are observed.

Another aspect to be considered when analyzing modes is the degree of dissociation they have between each other. This concept is extremely important in determining the severity of the patient’s pathology. The dissociation between modes in ST might be described in terms of structural organization of the personality and concerns the divisions and the organization of the personality or consciousness (i.e., structural dissociation), as originally advocated by Janet (1907). Dysfunctional schema modes are essentially ‘facets of the self’ that have not been integrated into a cohesive personality structure and therefore operate in a dissociated manner (Johnston et al., 2009). The constant alternation of the modes is directly related to their dissociated nature. The higher the dissociation between modes, the higher the emotional instability of the person. Moreover, the higher the dissociation between one mode and the others, along with the dissociation between modes and healthier aspects of the Self, the more they become increasingly maladaptive (Young et al., 2003). For example, some patients with Narcissistic PD show a constant activation of the *Self-Aggrandizer* mode. When alone, they activate the *Detached Self-soother* mode. These coping modes try to avoid contact between the subject and the *Lonely Child* mode. If the subject is aware and capable of accessing the latter mode, this is a sign of low levels of dissociation, meaning the subject understands his/her needs and how to satisfy them. Individuals that have a higher awareness of their modes’ way of functioning don’t show pathological symptoms, even if their personality structures are quite similar to ones seen in some PDs. Another problematic aspect of dissociation is when dysfunctional dissociated modes are integrated each other. In particular, for emotional dysregulation, when the *Impulsive Child* mode is associated with the *Abandoned and Abused Child* mode. In this case the trigger events are able to evocate a disruptive behavioral reaction and the person is not able to have an emotional control over behavior.

## A Strategy to Regulate Emotions

Based on the assumption we made in paragraph 2, *every Mode is associated with dysregulated emotional states or a dysregulatory strategies*, the therapist works with Modes, in order to foster emotion regulation. The overarching *strategy* and steps to regulate emotions in ST are the following: (1) Mode identification. If the patient experiences a dysregulated emotional state in the session (but also outside the session), the therapist tries to find out the Mode responsible for that state (for example, “Punitive Parent”). (2) Mode work. Once the Mode is recognized, the therapist uses a series of specific *techniques* to resolve that Mode (“Chair work” to fight the Punitive Parent). (3) Mode change. Once the Mode is deactivated, the experience of a more functional modality is facilitated (for example, the activation of the “Happy Adult”). As an effect of step 2, the patient experiences a down-regulation of negative emotions, and as an effect of step 3, he/she experiences an up-regulation of positive, self-soothing emotions. The techniques belonging to Steps 2 and 3 are different and depend on the Mode that is active in that moment. Every Mode is characterized by up- or down-regulation of specific emotions. When intervening, the clinician must monitor the presence of exaggerated or blunted emotions or even their apparent absence (say for example, an excessive distant and cold attitude of the patient). This can guide the clinician to understand which Mode is active in that moment (in this example, the Coping Mode “Detached Protector”). Sometimes the type of emotion is not sufficient to distinguish between Modes. The therapist has to also assess the way that emotion is expressed and its function. Some examples follow: Anger is an indicator of the *Angry Protector* mode or of the *Angry Child* mode. To disambiguate between the two, the clinician must observe the way anger is expressed. The *Angry Protector* mode is an avoidant coping style, aiming, for example, to keep the psychotherapist away from accessing certain experiences. During a psychotherapeutic session, the therapist may ask the patient to explore a specific traumatic life experience. If the *Angry Protector* mode is active, the patient may react in aggressive manner, saying for example: “Why do we have to talk about this bullshit all the time? It’s useless! You still can’t understand how I feel? What kind of therapist are you?” The patient usually feels fear to face certain traumatic memories that were not correctly elaborated because of their nature. In this way, this avoidance coping strategy prevents re-experiencing and re-elaborating these memories. Consider that the *Angry Protector* mode activates a sense of bewilderment, guilt or inadequacy in the therapist, sometimes even activating his/her coping strategies. The activation of the therapist’s coping strategy could be followed by a “dysfunctional interpersonal cycle.” The result of this vicious circle is that painful issues are pushed away from the session – this does not allow a further processing of these memories.

The *Angry Child*’s anger, instead, is reactive to frustration of a basic need. For example: the patient has an explosion of anger when the therapist arrives late to the session: “I’ve been waiting for 15 min, is this the care and attention you have for your patients? I wonder what the one before had to say! I knew I couldn’t trust you, I refuse to pay for the whole session!” In this case the anger is reactive to the frustration of a specific need: the need to be respected, seen and considered by the therapist. The *Angry Child*’s reaction might be understandable, if it were not so excessive. The patient’s anger is one of the few emotional strategies that the patient is able to use to meet his/her needs in this situation. The patient does not want to create a distance like in *Angry Protector* mode. If this mode is to be investigated, sadness is felt before anger, because the patient did not feel seen, heard or understood.

On the other hand, the anger of the *Bully and Attack* mode is a rage with the purpose of annihilating the person the patient is facing. This type of mode can be found in patients with severe PDs or forensic patients. This rage usually serves the purpose of ending the ongoing relationship, typically when the subject feels like his/her rights haven’t been respected. In this last case this mode is quite similar to the *Angry Child* mode, with the difference that the latter never actually harms others, since it reflects a need to be seen, not a need to break relationships.

Along with modes that share the same emotion, there are also modes that imply a deletion or modification of emotions. This is the case of the *Detached Protector* and the *Detached Self-soother* modes. Those two modes have the purpose of keeping the subject away from emotions. This doesn’t allow him/her to use emotions as a feedback, therefore hindering the comprehension of his/her needs.

A telltale sign to spot the *Detached Protector* mode is when, during the session, traumatic or strongly depriving life events are narrated without the subject showing any emotions about them. If asked to explain this lack of emotion, the patient usually answers with statements like: “Yes, it was very sad at the time. But it’s all over now.” When this mode activates the therapist usually feels boredom, detachment and coldness in the therapeutic relationship. Everything is filtered through rationality, the *Detached Protector*’s sharpest tool. This detachment doesn’t allow the patient to activate some incorrectly elaborated traumatic memories.

This protector is one of the most frequently seen coping modes in Borderline PD (BPD). BPD is one of the first PD on which ST efficacy has been tested (Giesen-Bloo et al., 2006). The *Detached Self-soother* mode has the *Detached Protector* mode’s same goal, i.e., to keep a safe distance from emotions and emotional needs. It reaches this objective by occupying the subject’s mind with repetitive activities. Subjects that report substanceless addiction (e.g., compulsive shopping, pathological gambling, Internet addiction, work addiction) show an active *Detached Self-soother* mode when they act out the addiction-related ritual. Compared to the *Detached Protector* mode, the *Detached Self-soother* mode also employs a finer strategy for emotional control. The former silences emotions with logic and emotional detachment. The latter, on the other hand, proposes a different emotion associated to the activity it uses to distract the patient’s mind from the feeling of vulnerability. As an example, a subject who has often felt loneliness and abandonment in childhood may try to stop the feeling of emptiness and sadness with pornographic material when alone. So, in this example, the *Detached Self-soother* mode replaces the negative feelings with sexual excitement.

## Techniques to Regulate Emotions

Once the Mode has been clearly detected (Step 1), the clinician may want to use one or more specific techniques designed to rework the active Mode. In this work, the main techniques are grouped in three main clusters (see **Tables 1–4**).

**Table 4:** Categories of dysregulated emotions in relation to modes and therapeutic strategies: functional modes.

Modes category	Dysregulated emotion	Dysfunctional regulation strategy	Effects	Therapeutic strategy
Healthy Adult	//	//	Nurturing Validation and affirmation of the child modes Parenting Taking responsibility and committing Pursue of pleasurable adult activities Health maintenance Athletic activities	The therapist helps the development of the Healthy adult using limited reparenting Validate emotional needs and emotions Protect the patient’s child mode from maladaptive modes Overcome dysfunctional coping modes
Happy Child	//	//	Love Satisfaction Fulfillment Nurturing Connection	Help to express emotions and needs using healthy ways to deal with emotions

(A) Cluster 1: *Relational techniques*. One of the major techniques used to convey emotional regulation during treatment is the relationship between the therapist and the patient. This set of techniques follow psychodynamic principles derived from object relation theory and Self psychology (Clarkin et al., 2007; Maroda, 2009). This kind of relationship is designed to contrast the abusive or punitive relationship that the patient experienced during his/her childhood. The therapy situation becomes a safe place in which the patient can affirm and express his/her needs, desires, and feelings. It also provides an emotional-relational corrective response (an extension of the emotional corrective response, Alexander and French, 1946). This kind of relationship helps the *Child* to experience and express feelings or desires that were banished or frustrated by the *Punitive Parent*. This corrective relationship is experienced inside most sessions as the therapist explores the present interpersonal difficulties of the patient. After the exploration of present life, the discussion goes to the childhood and adolescence; a link between present and past situations is made. During these explorations, the goal is to help the patient to access and elaborate the problematic child modes, thanks to the therapist’s figure limited reparenting (Kellogg and Young, 2006).
(1) *Limited reparenting*. Limited reparenting is the heart of ST (Behary and Dieckmann, 2013). The core concept of limited reparenting can be defined as a wide range of responses, behaviors, and attitudes of the therapist designed to respond to patient’s core needs (Arntz and Jacob, 2012). This process helps the patient recognize and meet unacknowledged needs from his/her early childhood, so that he/she can recognize and satisfy the present ones (Arntz, 2014). This experiential process is able to reach emotional structures of the brain such as the amygdala, in which emotional memories are stored (LeDoux, 1995). When an individual encounters negative stimuli or situations, such as the emotions, bodily sensations, and cognitions from his/her childhood, such as abandonment, abuse, neglect, or rejection, the amygdala is activated (Pynoos et al., 1999). The activation of the amygdala is very fast and automatic, occurring through memory channels (Phelps and LeDoux, 2005). Although the amygdala is connected with prefrontal areas, it is poorly affected by purely cognitive areas (Gray et al., 2002; Gerdes et al., 2010; Shiba et al., 2016). For this reason, these memories are hard to change by the simple activation of cognitive areas. In schema therapy the therapist helps the patient activate these memories, in order to satisfy the unmet needs related to these memories (Martin and Young, 2010). Emotional memories are then rewritten and changed. We hypothesize that this happens because of a memory reconsolidation process (Nader, 2003). When the patient reactivates past experiences, the neural system of the amygdala is no longer activated in the same way. After this, a positive alteration in behavioral and emotional response can be observed (Fink et al., 1996). The recognition and satisfaction of these needs in these situations, through different exercises and therapeutic relationship, allows the patient to create a restorative experience against those damaging experiences that have created his/her schemas and modes (Rafaeli et al., 2010). The core therapeutic process involved in limited reparenting is based on a secure attachment between therapist and patient. In fact, we know that the development of a healthy attachment is a prerequisite for normal psychological functioning. Limited reparenting uses the therapeutic relationship as a secure base that meets the deep needs that the patient has never seen fulfilled by his/her parents, such as security, stability, acceptance and autonomy. The first step of limited reparenting is to teach to patient how to regulate his/her emotions. This is done by using modeling techniques, in which the patient follows the therapist’s method of emotional regulation, within the limit of therapeutic framework. The emotional regulation carried out by the therapist will then be internalized by the patient and subsequently form a part of the *Healthy Adult* mode. The *Healthy Adult* is able to see his/her own needs, understand reality, and become autonomous. In fact, the main goal of the therapy is to teach the patient how to strengthen the *Healthy Adult* mode, giving more and more space to it, thanks to the internalization of the therapist’s healthy adult model. To maintain this, the therapist has to reach the *Vulnerable/Abused Child* mode to try and meet the needs of this mode with different psychotherapeutic instruments. Reaching the *Child* mode helps the therapist create a healthy and essential bond with the patient. Direct access to the *Vulnerable Child* is the cornerstone to give the therapist the possibility to meet those needs, and is the mainstay of treatment. This can only occur after a long process in which he/she has managed to overcome dysfunctional modes to reach the *Vulnerable Child* mode. Usually, in order to do this the therapist needs to confront and dismantle these dysfunctional coping modes. Limited reparenting simulates a real reparenting and may provide a patient with warmth, empathy, compassion, as well as firmness, reciprocity, respect for limits, and the recognition of the patient’s rights. Naturally, it also depends on the therapeutic stage that has been reached. Dealing with the dysfunctional coping modes is the first stage of therapy. Once the coping modes are overcome, both *Vulnerable Child* modes and *Punitive/Demanding Parent* modes will emerge. The second stage of therapy is to delete the punitive/demanding modes and to protect the *Vulnerable Child*. At this point, however, the interaction with the therapist has already been internalized; this is realized in *Healthy Adult* mode. In fact, the third stage of therapy is building a *Healthy Adult* mode. It is fair to say that in the therapeutic process of ST the “validation process” becomes the key step in all techniques used. The patient should never be perceived as wrong.
(2) *Empathic confrontation* is often used to deal with dysfunctional ways of coping. This technique allows a balance between the emotional validation of dysfunctional coping modes and the push for change. When the patient adopts dysfunctional patterns based on his/her schemas during a session, the therapist shows understanding toward him/her and stresses how those patterns derived from his/her childhood experiences. At the same time, however, the therapist draws attention to the fact that those patterns may very well not be accurate and that the patient’s behavior can lead to an unhappy life (empathic reality testing). Therefore, empathic confrontation requires a constant switching between empathy and reality check: the therapist validates schemas and coping styles as an understandable outcome of the life history of patient and at the same time brings his/her attention to their current negative consequences.
(B) Cluster 2*: Experiential techniques. Experiential techniques* are focused on emotions, and are designed to deal with emotions related to the activation of specific EMSs. They derive from emotion focused therapy (Greenberg, 2015), and take partial inspiration from the CBT tradition. These techniques give the patient the possibility to experience anger and sadness in a more adaptive way, or to build new systems of meaning and behaviors related to those emotions. In ST experiential techniques can be grouped into two main categories: imagery rescripting and dialog with the chairs. These techniques are then appropriately used depending on which mode the therapist is faced with, in the sessions.
(1) *Imagery rescripting.* In the *imagery rescripting* exercises, schemas and modes are activated with their associated unpleasant emotions. These emotions are related and connected to biographical traumatic memories (Marieke et al., 2011). The psychotherapist asks the patient to find in his/her imagination, through an emotional bridge, a situation in which, as a child, he/she experienced an emotion similar to the present negative emotion. In these exercises, the traumatic childhood experiences are changed, and acquire new meaning through the therapist’s support in the imagery. The therapist (or another adult protective person chosen by the patient) will take part at the scene and will help the patient in meeting the needs of the child. During imagery rescripting, negative emotions (anxiety, sadness, disgust, fear...) are changed, in the first instance thanks to the intervention of the therapist that, entering in the scene, is able to meet the need of the child (see **Table 5**). The fact that the patient’s needs are recognized and protected, in a situation where no one did it, creates two effects in the patient. The first is that the patient realizes that he/she deserves to be recognized and protected. The second is that the healing experience felt gives the patient a different viewpoint of that traumatic situation, a new possibility to experience similar situations in a safe way. With the continuation of treatment, the relationship with the therapist – and the modeling that is created during the imagery – creates a healthy pattern in the patient, an adult mode (i.e., internal working model), that is able to see the patient’s needs and to have a realistic and practical vision of reality. In fact, in a more advanced stage of therapy the patient’s *Healthy Adult* enters the scene and tries to meet these needs, during imagery rescripting exercises. It is often observed that when the patient returns to a past experience, in which he/she has felt a similar emotion to that of the present, he/she begins to understand the meaning of the emotion experienced in the present. The therapist enters the scene and takes care of the child patient, trying to meet his/her needs, allowing the patient to begin to recognize them, and figure out how to satisfy any of them, whatever they are; the subject needs to be seen, protected, unconditionally accepted, loved, validated and limited.
(2) *Chair Work*. In *chair-work* (Kellogg, 2004), dialogs between the patient’s different modes are conducted in order to help him/her to develop awareness about his/her mode activation and lack of integration. As a final step the chair-work leads to potentiate the *Healthy Adult* that should be able to contrast maladaptive modes, nurturing *Vulnerable Child* modes and let *Happy Child* express him/herself. For example, patients are supported to get in touch with feelings of anger and rage in the *Angry Child Demanding* and *Punitive Parent* modes, in *Healthy Adult*’s chair. The dialogs with the chairs, then, are good tools to deal with the coping modes. At the start of the trial the therapist underlines the importance of these modes, forming, in this way, the foundations for a more critical discussion. In fact, validating, comparing, limiting the dysfunctional coping modes, the therapist can make the patient more aware of the role that these modes have always had from childhood, how they developed, and then why the patient perceives them as ego-syntonic. It also allows seeing the disadvantages that these modes have today, in terms of negative interpersonal consequences. Subsequently, this work gives the opportunity to the patient to recognize them in everyday life, and allows him/her to limit the effect of these modes in his/her life. In chair-work, it is important that the patient enters fully into the mode’s perspective, and only speaks to the therapist from that perspective. In the same way, the therapist addresses the patient with the mode’s name, as if he/she were only speaking to this part of the patient. The chair-work is able to correct two different maladaptive phenomena in the patient’s mind: one is the dissociation, perceived in terms of process (i.e., breakdown in integrated processing; Van der Hart et al., 2006; Dorahy and Van der Hart, 2007; Steele et al., 2010), and the other is the excessive integration among different maladaptive modes. When a patient enters a dissociated mode, he/she can experience a wide array of experiences, from low dissociation to high dissociation state. In the former, the patient is able to remember different events or life themes related to different modes. In the latter case, the patient is not able to remember or to be aware about situations with different emotional value experienced in his life when other modes were active. In this way, the chair-work is able to enhance the metacognitive ability to recognize different modes, to be aware about it and about related memories.
Moreover, this exercise can also maximize the ability to differentiate different modes when they are integrated. Usually, we observed the co-activation of different modes, such as *Vulnerable Child* and *Punitive Parent*. In this case, the patient experienced strong feelings of guilt, vulnerability and sadness. With chair-work, the patient becomes aware about the fact that feelings of guilt are caused by a *Punitive Parent*, an internalization of past interactions with the caregiver that blames the *Vulnerable Child*, causing feelings of sadness and vulnerability. Having the opportunity to differentiate these modes is the first step to cope with them in a functional way.
(C) Cluster 3: *Cognitive techniques*. In ST the therapists also uses some CBT techniques, but only after the first stages of therapy are done. In fact, using CBT before having dismantled maladaptive coping strategies might reinforce them rather than reduce them. These techniques are derived from more standard CBT approaches and help the patient to cognitively understand their modes, coping strategies, function of emotions and to restructure eventual thinking patterns or break dysfunctional behavioral patterns. Writing flash cards, in which the patient report Modes activation and associated beliefs, and their effects, is an example.

**Table 5:** Imagery rescripting: steps of the process.

(1) Relaxation and creation of a safe space
(2) Accessing a difficult image from the present
(3) Creating an emotional bridge from the difficult present image
(4) Accessing a past image with a similar emotional correlate, focusing on needs and emotions of the child
(5) Introducing a figure who will care for the needs of that child (therapist or healthy adult), so that the situation may change
(6) Stabilize a sense of security and positive attachment
(7) Translate the new emotional meaning to the initial situation

Although some parallels can be made between ST strategies and techniques and Gross cognitive model of emotion regulation (CER) (see Fassbinder et al., 2016), we believe a Dynamic-Experiental model of Emotion Regulation model (EDER, Grecucci et al., 2015b, in press) may better fit ST methodology. According to the CER model, emotions are generated according to a precise sequence in which an individual exposed to a situation: (1) engages it; (2) attends to a particular aspect of the situation; (3) interprets the event; (4) experiences an emotional response with a behavioral (action tendency), emotional, and physiological arousal; and (5) modulates that response. Following this model, emotion regulation or dysregulation can happen at any step in this sequence and every emotion can become dysregulated. The main mechanism of dysregulation is the lack of, or failure to apply, an appropriate regulatory strategy. Cognitive Behavior Therapy (Beck and Fernandez, 1998), and Dialectical Behavior Therapy (Linehan, 1993) use interventions for emotional regulation that fit with CER model. Within this model and these therapies, emotion dysregulation is treated through behavioral methods, attentional methods, cognitive methods and mindfulness and acceptance methods. The Experiential-Dynamic Emotion Regulation model (Grecucci et al., 2015b, in press; see also Campos et al., 2004 for a similar account) claims that events trigger: innate emotional responses with inborn adaptive action tendencies which precede cognition (temporal and neuroanatomical primacy) (Grecucci et al., 2016). Once elicited, emotions have a duration and intensity proportional to the stimulus and automatically self-regulate. The conscious control or regulation is therefore not required. Emotions are generated, expressed, and channeled into healthy actions and automatically return to baseline. Thus, emotions are not inherently dysregulated. Dysregulation derives from the combination of emotions plus conditioned anxiety, or of emotions with a dysregulatory strategy (for example, sadness for failing in an exam plus the intervention of a maladaptive Parent mode that creates shame, guilt, and contempt toward the self; in psychodynamic terms, a defense mechanism of self-attack) leading to dysregulated emotional states. To regulate these states, the clinician must remove the pathological Modes. Once removed, automatic emotion regulation follows. For this reason, ST rarely teaches explicit regulatory strategies (such as in CBT or DBT), but works on the underlying cause that creates the observed dysregulation.

## Application

We are going to present a clinical case to give an example of strategies and techniques used in ST for the treatment of emotional dysregulation. When the therapist^1^ met the 36 year old Linda in May 2014, she had the impression of having a sad, impulsive, angry and emotionally unstable woman in front of her. She was also 15 min late. Linda had decided to see a therapist because she was suffering from strong mood swings, fits of anger, agitation, central insomnia characterized by waking up frequently and anhedonia. These symptoms had taken a turn for the worse after the end of the relationship with her boyfriend, with whom she had been for 19 years.

Linda says “I’ve always been a bit moody, sometimes I feel good around people, other times I argue furiously. I don’t know what’s happening to me,” “When someone criticizes me I can’t stay quiet. I get really angry, I feel a heat surging from my chest and just coming out!” Linda grew up in an environment that ignored every need for care, affection, attention, listening, and understanding. Her parents were often absent, physically and emotionally.

As a consequence of her personal life Linda shows, as is the case with many BPD patients, several EMSs: Mistrust/Abuse, Abandonment/Instability, Emotional Deprivation, Defectiveness/Shame, Failure, Self-sacrifice, and Unrelenting Standards. The first 4 EMSs we mentioned imply that the needs for safety, nurture, empathy and security were not adequately satisfied, Failure implies that the patient lives with strong feelings of guilt for not meeting her family’s exaggerated expectations. Thus, the last 2 EMSs (Self Sacrifice and Unrelenting Standards) are developed: both are based on an excessive focus on the desires and needs of others, at the expense of personal needs.

The ST therapist must always try to validate the patient’s experience, so in this first phase no action must be taken to restructure the processes behind emotional dysregulation: the focus is on validation. Therefore, instead of working on EMSs, the first step in Linda’s therapy was focusing on modes that emerged during sessions. The patient lived strong emotions that changed in just a few seconds. This is typical in cases of BPD. For example, when talking about her affective relationship, Linda feels a terrible need to open up and feel loved for the person she is. At the same time, she’s terrified of showing herself, because in her experience she has always been criticized and judged by her parents in a very negative way, regardless of what she did.

To highlight modes, the therapist utilized the Schema Mode Inventory (SMI, Young et al., 2007) and ecological observation of what happened during the session, paying special attention to the emotions that emerged and their somatic manifestations. For example, when Linda claims to be very angry, the therapist asks: “*How does this emotion make you feel?*”, “*Where do you feel it on your body?*”, “*What do you do to manage it?*” “*Where is it coming from, what triggered it?*”, “*How do you feel afterward?*” By doing this, the following modes have been identified: Detached Protector, Abandoned and Abused Child, Enraged Child, Punitive Parent and the Healthy Adult, although the latter was very weak.

When Linda feels she’s being criticized by an external agent, the Punitive Parent mode, along with the Abandoned and Abused Child mode, are activated. Those two modes are deeply set in Linda’s personality. This makes Linda feel like she is profoundly wrong, inadequate, inferior, unworthy of other people’s love. When this happens, the Enraged Child comes out, only to be inhibited by the strong Detached Protector. This mode does not allow her to feel any emotions, making her act like a robot and making her avoid situations that trigger these emotions. A strong sense of derealization is seen in this mode, reality appears to be muffled. If the protector doesn’t act, a strong rage is triggered, and Linda vents all her frustration verbally and physically, sometimes throwing objects. This happens when criticism is seen as something final and unchangeable.

During therapy the patient was taught how to identify and recognize different modes and needs, while also being taught that modes are an adaptive response to attachment needs that have not been satisfied in childhood, adolescence, and the current period. In order to effectively give Linda this knowledge, the therapist openly asked her questions about her childhood and adolescence, maintaining as much eye contact as possible, showing sincere interest for her life story, and validating her emotional experiences.

The only moment in which the patient does not receive validation is when the Punitive Parent emerges: in this case, the therapist makes the patient notice how this part is based on interiorized negative experiences, how this part does not belong to her. Thus, the therapist asks “*Does Linda really need to feel like this?*”, “*Is this really what Linda thinks about herself?*”

The third phase in therapy is designed to modify emotional dysregulation with limited reparenting: the therapist uses this technique to pose as a new safe and accepting attachment figure, so that a new, healthy operative model can be created for Linda.

By doing this, Linda started getting in touch with her emotions, and started, albeit with much effort, labeling different parts of her with names like “Small Linda,” “Angry Linda,” “Warrior Linda,” “Top-of-the-class Linda”: respectively the Abandoned and Abused Child, the Enraged Child, the Detached Protector and the Demanding Parent.

With limited reparenting the therapist tries to satisfy the patient’s emotional needs by giving warmth, nurturing and care, being truthful, honest and straightforward, empathizing with the patient and validating his/her emotional states and feelings. To put it briefly, the therapist acts as a model for a healthy adult by behaving like a healthy adult that satisfies a child’s needs. For example, when Linda started sessions in a detached and cold way, the therapist made her notice that the Detached Protector mode was activated. To bypass it and reach the Abandoned and Abused Child, chairwork was used. The therapist made the patient sit on the Warrior Linda chair and told her “*I realize that right now Warrior Linda is here with us, and that she’s trying to protect you. I would like to thank her for protecting you, I know how painful it is for you to talk about these things, so I understand that a part of you tries to protect you from feeling that deep sadness and that sense of emptiness and abandonment again. I know your life, I know what happened to you, you were a small child and you should have had somebody who took care of you. Unfortunately, nobody was there. I remember when you told me about that time your father was hitting you and your mother was standing there, looking at you without doing or saying anything. No child can bear this anguish, this fear, you had to find a way to avoid feeling it, and Warrior Linda helped you with this. It’s normal, and I understand that even now that there’s a person who wants to help you and take care of you Warrior Linda is trying to protect you and to keep emotions hidden. But exactly because I understand what you felt, I have to ask Warrior Linda to let me talk with the part that is suffering, so that I can ask what she needs.*”

Once contact has been made with the Abandoned and Abused Child, the therapist starts a rescripting exercise that involves reparenting. Linda has to close her eyes to visualize her safe space, where her tolerance to emotions is heightened. By doing this, the triggering of dissociative mechanisms is avoided. When the therapist sees that Linda is in a stable and relaxed mood, she asks her to visualize the past situation that triggered those strong emotions. Once the situation is visualized, the therapist focuses on what Linda is feeling, allowing her to feel what she needs and the related emotion. Emotion is used as a catalyst for childhood situations in which Linda has felt a similar emotion. At this point, the therapist asks Linda where she is, how old she is, what is happening, what she looks like, and how she feels. The patient starts to slowly take on the facial expression of a frightened child, her voice has changed, it’s softer, and she whispers what she sees behind her eyelids. In this case, the Little Linda mode is fully accessible. The therapist validates the emotions that the child feels, and uses rescripting to stop any aggression, so that Linda can know what being protected feels like.

Once Linda’s need for a sense of safety, genuine interest, and value is satisfied, the therapist takes her back to the initial scene. Now that Linda has felt protection and care, she does not feel wrong anymore; instead, she sees her needs and acts to satisfy them in a functional way.

This work is at the roots of the development of a new relationship, based on trust and attachment with a healthy figure that sees needs and acts to satisfy them, validating the patient’s emotions. In this way, the patient interiorizes a new, healthy model for a relationship. In a more advanced therapeutic phase these imaginative exercises are designed to allow the adult patient to intervene, so that she can protect and validate the needs of her child Self.

This is an important passage in the path of consolidating the internal operational model of the Healthy Adult. The patient therefore becomes capable of recognizing her emotions, of connecting them to her childhood experiences, of expressing and satisfying her needs in the present, self-regulating her emotions in an adaptive way. This happens because Adult Linda puts limits to Angry Linda’s behavior and excludes Top-of-the-class Linda and Warrior Linda, while creating an emotional connection to Small Linda’s needs. During therapy the patient often jumps from one mode to the other very rapidly (i.e., flipping). This often begins when a critical or hostile mode emerges, such as Top-of-the-class Linda, that makes Small Linda feel wrong and alone. This emotional state activates Angry Linda, in order to avoid feeling the sadness and sense of powerlessness that Small Linda brings. However, this mode inevitably reactivates Top-of-the-class Linda, and Little Linda is humiliated again: *“See? You’re getting angry again, you’re the same old spoilt brat!”* In order to break this vicious cycle, Warrior Linda is activated and muffles all emotions. The therapist uses chair-work to make Linda notice how all these parts activate, what triggers them, how long each part is active for. This work has the purpose of enhancing the patient’s awareness of these different parts by acting on her meta-cognitive capability. Another focal point of chair-work is working on the Top-of-the-class Linda mode, stripping it of legitimacy. Top-of-the-class Linda says: “*See? Small Linda is wrong; she’ll never be able to be like the others.*” The therapist answers: “*Here’s Top-of-the-class Linda, always trying to convince you that you’re not like the others, that you’re wrong, but we now that this is a useless part that doesn’t see what you really need. I think we should send her away immediately, you don’t deserve to sit here and believe what she says.*”

By weakening the punishing part, the integration between the Healthy Adult and the Abandoned and Abused Child is strengthened. The patient manages to recognize the punishing part and shut it out, letting the healthy part that recognizes her emotions and needs talk. This constant co-activation of her vulnerable part and her healthy part enhances her emotional regulation capability.

During therapy the patient says she feels connected to Small Linda, that she needs to protect her and listen to her real life needs and necessities, acting like a Healthy Adult. Linda has often referred effort and difficulty in having to take on negative emotions.

One of the fundamental parts of this therapy has been the therapeutic relationship. The therapist has always been sincere, straightforward, and empathetic. This has allowed Linda to experiment what has probably been her first healthy relationship, along with allowing her to trust the therapist and show her vulnerable side, in order to share it and work with it through mode work.

Sessions lasted for 10 months: twice a week for the first 6 months, once a week for the last 4 months. At the end of this therapy, Linda shows a high level of tolerance for situations that used to trigger dysfunctional modes and emotions. The patient also managed to feel emotionally involved with people who know how to give her what she needs and that don’t constantly criticize her. She has also learned to make difficult choices, reducing her fear of abandonment and tolerating negative feedback. She doesn’t feel the presence of Top-of-the-class Linda when she relates to others, at least not as much as she used to. All the symptoms that brought her to start a therapy are gone. At the time being, her level of social functioning has allowed her to meet friends and have a relationship of healthy sharing with them. She has also started a new relationship, in which she feels loved and seen.

## Empirical Evidence And Future Direction

In the last decades, ST efficacy has been tested in different studies. Giesen-Bloo et al. (2006) using a multicenter randomized control trial, compared ST with Transference Focused Psychotherapy (TFP), a psychodynamically based psychotherapy. In this paper, the researchers tested the efficacy of ST compared to TFP on a population of patients with BPD. After only 12 months of treatment, ST showed its effectiveness in reducing BPD symptoms. After 3 years of treatment, ST showed to be superior to TFP in some of the measures. In Van Asselt et al. (2007) evaluated the cost-effective ratio between ST and TFP, finding that ST dominates over TFP in many items. In particular, logistic regression analysis with the treatment group and BPD baseline score as covariates showed a significant effect in favor of ST. Societal and informal care costs in the ST patients were lower and recovery rate was higher compared with the TFP group. In addition, another important result was replicated in this study: the proportion of patients who had recovered after 4 years was 52% for the ST group and 29% for the TFP group. From 2008 ST has been recommended as one of the evidence based treatments in the Dutch Guidelines on Personality Disorders (2008), and insurance companies reimburse for treatment. After this date the efficacy of ST has been demonstrated also in other PDs. Bamelis et al. (2014), with a multicenter Randomized Controlled Trial (RCT) design compared the ST with Treatment-as-Usual (TAU) and Clarification-Oriented Psychotherapy (COP) in cluster C, paranoid, histrionic, and narcissistic PDs. This study lasted 3 years and was conducted on 323 patients. All analyses consistently revealed that ST was superior to other treatments on greater recovery from PDs, as well as when recovery was defined more stringently, and when controlled for assessment instrument. Moreover, the lower dropout rate in ST suggests higher acceptability by patients. The number of patients still in treatment after 3 years was lowest in the ST group (13% vs. 26% in TAU and 36.6% in COP), pointing to the ability of ST to achieve at least comparable results in less time. An adjacent qualitative study assessing patient and therapist perspectives on ST (Bamelis et al., 2014) revealed that working with the mode model was highly appreciated by patients and therapists since it guided therapists in choosing adequate techniques and helped patients to better understand their own behaviors and feelings. Parallel with these studies, some studies have compared the effectiveness of ST with TAU in group therapy, on patients with BPD (Farrell et al., 2009). In particular, in this study Farrell et al. (2009) observed from 2 to 3 months into the 8 months of treatment not only meaningful reductions in impulsive, self-injurious behavior or loneliness and emptiness but also an increase in mood, affective features, quality of life issues, and global functioning. Nadort et al. (2009) demonstrated that ST efficacy in recovering from six types of PDs (avoidant, dependent, obsessive–compulsive, paranoid, histrionic, narcissistic). Its effect was superior to TAU and COP. ST had fewer dropouts, and superior cost-effectiveness. A randomized controlled trial comparing ST for forensic patients with PDs to usual treatment suggests strong effects of ST even in patients with high psychopathic traits (Bernstein et al., 2012). In these last years the research on the effectiveness of ST moved from PDs to other disorders like depression. In these studies, researchers using a single case series study design (Renner et al., 2013, 2016; Renner, 2014; Porter et al., 2016) or using RCT (Carter et al., 2013) showed that ST is not less effective than CBT. Studies have been conducted to test not only the effectiveness of ST, but also the effectiveness of specific techniques used in ST. In particular, some imaginative techniques, like imagery rescripting, were evaluated in several disorders or conditions (Grunert et al., 2007; Arntz, 2011; Stopa, 2011). Although ST was shown to be at least equal and for some measurements, if not superior to other types of therapies (Perry et al., 1999; Leichsenring and Leibing, 2003), we do not have an empirical demonstration of its power in regulating emotions yet.

## Conclusion

The literature up to now indicates that ST is an effective treatment for BPD. We believe ST efficacy is due to the structural change in the patient’s personality that every ST therapist aims to, and not only to symptomatic improvements. As a result of the structural changes, the initial emotional dysregulation due to maladaptive regulatory strategies (pathological Modes), gives way to (adult) emotional regulation. We think that the features of ST and the need of new treatments, that are able to bring about a full recovery for patients, will be a major propulsive boost in exploring new clinical applications of this model. This is quite probable, not only regarding ST’s effectiveness, but also regarding what is effective in ST and if it can be further enhanced to better understand and treat various ailments. Along this paper we provided theoretical and clinical implication of ST as a way of treating emotional dysregulation in a wide range of patients. Indeed, ST gives the therapist a set of instruments and techniques to foster emotional regulation through the therapeutic relationship and experiential emotion focused methods. Future studies will test this fascinating hypothesis.

## Author Contributions

HD, AG, and MP contributed equally to this work. HD, AG, and AC substantially contributed in the conception of the work. HD, EU, and IG drafted the work. HD, AG, and MP give the final approval of the work. HD, AG, and MP agree to be accountable for all aspect.

## Conflict of Interest Statement

The authors declare that the research was conducted in the absence of any commercial or financial relationships that could be construed as a potential conflict of interest.

## Footnotes

1 Also one of the article’s authors.

## References

[B1] Alexander F. G. French T. M. (1946). *Psychoanalytic Therapy: Principles and Applications*. New York, NY: Ronald, 10.1086/219999

[B2] Arntz A. (2011). Imagery rescripting for personality disorders. *Cogn. Behav. Pract.* 18 466–481. 10.1016/j.cbpra.2011.04.006

[B3] Arntz A. (2014). “Imagery rescripting for personality disorders: healing early maladaptive schemas,” in *Working with Emotion in Cognitive-behavioral Therapy: Techniques for Clinical Practice*, eds Thoma N. C. McKay D. (New York, NY: Guilford Publications), 175–202.

[B4] Arntz A. Bernstein D. P. Jacob J. (2012). *Schema Therapy in Practice.* Chichester: Wiley-Blackwell.

[B5] Arntz A. Jacob G. (2012). *Schema Therapy in Practice: An Introductory Guide to the Schema Mode Approach*. Chichester: John Wiley & Sons.

[B6] Bamelis L. L. M. Evers S. M. M. A. Spinhoven P. Arntz A. (2014). Results of a multicenter randomized controlled trial of the clinical effectiveness of schema therapy for personality disorders. *Am. J. Psychiatry* 171 305–322. 10.1176/appi.ajp.2013.12040518 24322378

[B7] Beck A. T. Emery G. (1985). *Anxiety Disorders and Phobias: a Cognitive Approach*. New York, NY: Basic Books, 10.1017/s0021932000016369

[B8] Beck R. Fernandez E. (1998). Cognitive-behavioral therapy in the treatment of anger: a meta-analysis. *Cogn. Ther. Res.* 22 63–74. 10.1023/A:1018763902991

[B9] Behary W. T. Dieckmann E. (2013). “Schema therapy for pathological narcissism: the art of adaptive reparenting,” in *Understanding and Treating Pathological Narcissism*, ed. Ogrodniczuk J. S. (Washington, DC: American Psychological Association), 285–300. 10.1037/14041-017

[B10] Bernstein D. P. Nijman H. Karos K. Keulen-de Vos M. de Vogel V. Lucker T. (2012). Schema therapy for forensic patients with personality disorders: design and preliminary findings of multicenter randomized clinical trial in the Netherlands. *Int. J. Forensic Ment. Health* 11 312–324. 10.1080/14999013.2012.746757

[B11] Bowlby J. (1969). *Attachment and Loss: Attachment*. New York, NY: Basic Books.

[B12] Burke A. Heuer F. Reisberg D. (1992). Remembering emotional events. *Mem. Cogn.* 20 277–290. 10.3758/BF03199665 1508053

[B13] Campos J. J. Frankel C. B. Camras L. (2004). On the nature of emotion regulation. *Child Dev.* 75 377–394. 10.1111/j.1467-8624.2004.00681.x 15056194

[B14] Carr S. N. Francis A. J. P. (2010). Do early maladaptive schemas mediate the relationship between childhood experiences and avoidant personality disorder features? A preliminary investigation in a non-clinical sample. *Cogn. Ther. Res.* 34 343–358. 10.1007/s10608-009-9250-1

[B15] Carter J. D. McIntosh V. V. Jordan J. Porter R. J. Frampton C. M. Joyce P. R. (2013). Psychotherapy for depression: a randomized clinical trial comparing schema therapy and cognitive behaviour therapy. *J. Affect. Disord.* 151 500–505. 10.1016/j.jad.2013.06.034 23870427

[B16] Christianson S. A. Engelberg E. (1999). “Organization of emotional memories,” in *Handbook of Cognition and Emotion*, eds Dalgleish T. Power M. (Chicchester: John Wiley and Sons), 211–229. 10.1002/0470013494.ch11

[B17] Clarkin J. F. Yeomans F. E. Kernberg O. F. (2007). *Psychotherapy for Borderline Personality: Focusing on Object Relations*. Washington, DC: American Psychiatric Press.

[B18] Conway M. A. Pleydell-Pearce C. W. (2000). The construction of autobiographical memories in the self memory system. *Psychol. Rev.* 107 261–288. 10.1037//0033-295X.107.2.261 10789197

[B19] Dorahy M. J. Van der Hart O. (2007). “Trauma and dissociation: An historical perspective,” in *Traumatic Dissociation: Neurobiology and Treatment*, eds Vermetten E. Dorahy M. J. Spiegel D. (Arlington, VA: American Psychiatric Publishing), 3–30.

[B20] Dutch Guidelines on Personality Disorders (2008). *Multidisciplinaire Richtlijn Persoonlijkheidsstoornissen. (Dutch Guidelines on Personality Disorders)*. Utrecht: Trimbos Instituut.

[B21] Farrell J. M. Shawn Ida A. Webber M. A. (2009). A schema-focused approach to group psychotherapy for outpatients with borderline personality disorder: a randomized controlled trial. *J. Behav. Ther. Exp. Psychiatry* 40 317–328. 10.1016/j.jbtep.2009.01.002 19176222

[B22] Fassbinder E. Schweiger U. Martius D. Brand-de Wilde O. Arntz A. (2016). Emotion regulation in schema therapy and dialectical behavior therapy. *Front. Psychol.* 7:1373 10.3389/fpsyg.2016.01373 PMC5021701 27683567

[B23] Field N. P. Horowitz M. J. (1998). Applying an empty-chair monologue paradigm to examine unresolved grief. *Psychiatry* 61 279–287. 10.1080/00332747.1998.11024840 9919623

[B24] Fink G. R. Markowitsch H. J. Reinkemeier M. Bruckbauer T. Kessler J. Heiss W. D. (1996). Cerebral representation of one’s own past: neural networks involved in autobiographical memory. *J. Neurosci.* 16 4275–4282. 8753888 10.1523/JNEUROSCI.16-13-04275.1996 PMC6579004

[B25] Fonagy P. (1998). Attachment theory approach to treatment of the difficult patient. *Bull. Menninger Clin.* 62 147–169. 9604514

[B26] Frankel M. (1993). Adult reconstruction of childhood events in the multiple personality literature. *Am. J. Psychiatry* 150 954–958. 10.1176/ajp.150.6.954 8494076

[B27] Freeman A. (1981). “Dream and images in cognitive therapy,” in *New Directions in Cognitive Therapy*, ed. Bedrosian R. C. (New York, NY: Guilford Press), 224–237.

[B28] Freud S. (1917). “Mourning and melancholia,” in *The Standard Edition of the Complete Psychological Works of Sigmund Freud*, Vol. 14 ed. Stratchey J. (London: Hogarth), 237–260. 10.1037/e417472005-352

[B29] Gerdes A. B. Gerdes A. Wieser M. J. Mühlberger A. Weyers P. Alpers G. W. (2010). Brain activations to emotional pictures are differentially associated with valence and arousal ratings. *Front. Hum. Neurosci.* 4:175 10.3389/fnhum.2010.00175 PMC2982745 21088708

[B30] Giesen-Bloo J. van Dyck R. Spinhoven P. van Tilburg W. Dirksen C. van Asselt T. (2006). Outpatient psychotherapy for borderline personality disorder: randomized trial of schema-focused therapy vs transference-focused psychotherapy. *Arch. Gen. Psychiatry J.* 63 649–658. 10.1001/archpsyc.63.6.649 16754838

[B31] Gray J. R. Braver T. S. Raichle M. E. (2002). Integration of emotion and cognition in the lateral prefrontal cortex. *Proc. Natl. Acad. Sci. U.S.A.* 99 4115–4120. 10.1073/pnas.062381899 11904454 PMC122657

[B32] Grecucci A. De Pisapia N. Venuti P. Palladino M. P. Job R. (2015a). Baseline and strategic mindful regulation: behavioral and physiological investigation. *PLoS ONE* 10:e0116541 10.1371/journal.pone.0116541 PMC4295876 25590627

[B33] Grecucci A. Di Marzio F. Frederickson J. Job R. (2016). “Anxiety and its regulation: neural mechanisms and regulation techniques according to the experiential-dynamic approach,” in *Anxiety Disorders*, ed. Durbano F. (Rijeka: InTech publishing).

[B34] Grecucci A. Frederickson J. Job R. (in press) How dare you not recognize the role of my contempt: insights from Experimental Psychopathology. *Behav. Brain Sci.* 10.1017/S0140525X16000777 29122033

[B35] Grecucci A. Giorgetta C. Bonini N. Sanfey A. (2013). Living emotions, avoiding emotions: behavioral investigation of the regulation of socially driven emotions. *Front. Psychol.* 3:616 10.3389/fpsyg.2012.00616 PMC3552385 23349645

[B36] Grecucci A. Job R. (2015). Rethinking reappraisal: insights from affective neuroscience. *Behav. Brain Sci.* 38:e102 10.1017/s0140525x14001538 26786023

[B37] Grecucci A. Thneuick A. Frederickson J. Job R. (2015b). “Mechanisms of social emotion regulation: from neuroscience to psychotherapy,” in *Handbook on Emotion Regulation: Processes, Cognitive Effects and Social Consequences*, ed. Bryant M. L. (New York, NY: Nova Publishing), 57–84.

[B38] Greenberg L. S. (2015). *Emotion Focused Therapy. Coaching Clients to Work Through Their Feeling.* Washington, DC: American Psychological Association.

[B39] Greenberg L. S. Safran J. (1984). Integrating affect and cognition: a perspective on the process of therapeutic change. *Cogn. Ther. Res.* 8 559–578. 10.4135/9781412973496.d25

[B40] Greenberg L. S. Safran J. D. (1989). Emotion in psychotherapy. *Am. Psychol.* 44 19–68. 10.1037/0003-066x.44.1.19 2930052

[B41] Gross J. J. (ed.) (2011). *Handbook of Emotion Regulation.* New York, NY: Guilford publications.

[B42] Grunert B. K. Weis J. M. Smucker M. R. Christianson H. F. (2007). Imagery rescripting and reprocessing therapy after failed prolonged exposure for post-traumatic stress disorder following industrial injury. *J. Behav. Ther. Exp. Psychiatry* 38 317–328. 10.1016/j.jbtep.2007.10.005 18037391

[B43] Guntrip H. (1995). *Personality Structure and Human Interaction: The Developing Synthesis of Psychodynamic Theory*. Londra: Karnac Books.

[B44] Holmes E. A. Arntz A. Smucker M. R. (2007). Imagery rescripting in cognitive behaviour therapy: images, treatment techniques and outcomes. *J. Behav. Ther. Exp. Psychiatry* 38 297–305. 10.1016/j.jbtep.2007.10.007 18035331

[B45] Janet P. (1907). *The Major Symptoms of Hysteria.* New York, NY: Classics of Psychiatry & Behavioral Sciences Library, Division of Gryphon Editions.

[B46] John O. P. Gross J. J. (2004). Healthy and unhealthy emotion regulation : personality processes, individual differences, and life span development. *J. Pers.* 72 1301–1333. 10.1111/j.1467-6494.2004.00298.x 15509284

[B47] Johnston C. Dorahy M. J. Courtney D. Bayles T. O’Kane M. (2009). Dysfunctional schema modes, childhood trauma and dissociation in borderline personality disorder. *J. Behav. Ther. Exp. Psychiatry* 40 248–255. 10.1016/j.jbtep.2008.12.002 19124119

[B48] Kellogg S. H. (2004). Dialogical encounters: Contemporary perspectives on “chairwork” in psychotherapy. *Psychotherapy* 41 310–320. 10.1037/0033-3204.41.3.310

[B49] Kellogg S. H. Young J. E. (2006). Schema therapy for borderline personality disorder. *J. Clin. Psychol.* 62 445–458. 10.1002/jclp.20240 16470629

[B50] LeDoux J. E. (1995). Emotion: clues from the brain. *Annu. Rev. Psychol.* 46 209–230. 10.1146/annurev.ps.46.020195.001233 7872730

[B51] Leichsenring F. Leibing E. (2003). The effectiveness of psychodynamic therapy and cognitive behavior therapy in the treatment of personality disorders: a meta-analysis. *Am. J. Psychiatry* 160 1223–1232. 10.1176/appi.ajp.160.7.1223 12832233

[B52] Linehan M. (1993). *Cognitive-behavioral Treatment of Borderline Personality Disorder.* New York, NY: Guilford Press.

[B53] Lobbestael J. Arntz A. Löbbes A. Cima M. (2009). A comparative study of patients and therapists’ reports of schema modes. *J. Behav. Ther. Exp. Psychiatry* 40 571–579. 10.1016/j.jbtep.2009.08.001 19698937

[B54] Lobbestael J. Arntz A. Sieswerda S. (2005). Schema modes and childhood abuse in borderline and antisocial personality disorders. *J. Behav. Exp. Psychiatry* 36 240–253. 10.1016/j.jbtep.2005.05.006 15953584

[B55] Lobbestael J. van Vreeswijk M. Spinhoven P. Schouten E. Arntz A. (2010). Reliability and validity of the short Schema Mode Inventory (SMI). *Behav. Cogn. Psychother.* 38 437–458. 10.1017/S1352465810000226 20487590

[B56] Lobbestael J. Van Vreeswijk M. F. Arntz A. (2007). Shedding light on schema modes: a clarification on the mode concept and its current research status. *Neth. J. Psychol.* 63 76–85. 10.1007/BF03061068

[B57] Lobbestael J. van Vreeswijk M. F. Arntz A. (2008). An empirical test of schema mode conceptualisations in personality disorders. *Behav. Res. Ther.* 46 854–860. 10.1016/j.brat.2008.03.006 18460405

[B58] Marieke C. Abma T. A. Bamelis L. Arntz A. (2011). Personality disorder patients’ perspectives on the introduction of imagery within schema therapy: a qualitative study of patients’ experiences. *Cogn. Behav. Pract.* 18 482–490. 10.1016/j.cbpra.2011.04.005

[B59] Maroda K. J. (2009). *Psychodynamic Techniques: Working with Emotion in the Therapeutic Relationship*. New York, NY: The Guilford Press.

[B60] Martin R. Young J. (2010). “Schema therapy,” in *Handbook of Cognitive-behavioral Therapies*, ed. Dobson K. S. (New York, NY: The Guilford Press), 317–347.

[B61] Nader K. (2003). Memory traces unbound. *Trends Neurosci.* 26 65–72. 10.1016/S0166-2236(02)00042-5 12536129

[B62] Nadort M. Arntz A. Smit J. H. Giesen-Bloo J. Eikelenboom M. Spinhoven P. (2009). Implementation of outpatient schema therapy for borderline personality disorder with versus without crisis support by the therapist outside office hours: a randomized trial. *Behav. Res. Ther.* 47 961–973. 10.1016/j.brat.2009.07.013 19698939

[B63] Pedersen S. H. Poulsen S. Lunn S. (2014). Affect regulation: Holding, containing and mirroring. *Int. J. Psychoanal.* 95 843–864. 10.1111/1745-8315.12205 25351730

[B64] Perls F. S. Baumgardner P. (1975). *Gifts from Lake Cowichan: Legacy from Fritz*. Palo Alto, CA: Science and Behavior Books.

[B65] Perry J. C. Banon E. Ianni F. (1999). Effectiveness of psychotherapy for personality disorders. *Am. J. Psychiatry* 156 1312–1321. 10484939 10.1176/ajp.156.9.1312

[B66] Phelps E. A. LeDoux J. E. (2005). Contributions of the amygdala to emotion processing: from animal models to human behavior. *Neuron* 48 175–187. 10.1016/j.neuron.2005.09.025 16242399

[B67] Pope K. S. Singer J. L. (eds) (1978). *The Stream of Consciousness.* New York, NY: Plenum Publishing Corporation, 10.1007/978-1-4684-2466-9

[B68] Porter R. J. Bourke C. Carter J. D. Douglas K. M. McIntosh W. Jordan J. (2016). No change in neuropsychological dysfunction or emotional processing during treatment of major depression with cognitive–behaviour therapy or schema therapy. *Psychol. Med.* 46 393–404. 10.1017/S0033291715001907 26446709

[B69] Pynoos R. S. Steinberg A. M. Piacentini J. C. (1999). A developmental psychopathology model of childhood traumatic stress and intersection with anxiety disorders. *Biol. Psychiatry* 46 1542–1554. 10.1016/S0006-3223(99)00262-0 10599482

[B70] Rafaeli E. Bernstein D. P. Young J. (2010). “The therapeutic relationship: limited reparenting,” in *Schema Therapy: Distinctive Features*, ed. Dryden W. (New York, NY: Routledge), 153–157.

[B71] Renner F. (2014). Treatment for chronic depression using schema therapy. *Verhaltenstherapie* 24 169–181. 10.1111/cpsp.12032

[B72] Renner F. Arntz A. Leeuw I. Huibers M. (2013). Treatment for chronic depression using schema therapy. *Clin. Psychol.* 20 166–180. 10.1111/cpsp.12032

[B73] Renner F. Arntz A. Peeters F. P. M. L. Lobbestael J. Huibers M. J. H. (2016). Schema therapy for chronic depression: results of a multiple single case series. *J. Behav. Ther. Exp. Psychiatry* 51 66–73. 10.1016/j.jbtep.2015.12.001 26780673

[B74] Rijkeboer M. M. Huntjens R. J. C. (2007). *EMS’s in Forensic Settings: An Implicit Measure of Insufficiënt Self-Control/Self-Discipline. A Pilot Study*. Paper presented World Congress of Behavioural & Cognitive Therapies, Barcelona

[B75] Safran J. D. Greenberg L. S. Rice L. N. (1988). Integrating psychotherapy research and practice: Modeling the change process. *Psychotherapy* 25 1–17. 10.1037/h0085305

[B76] Samuels M. Samuels N. (1975). *Seeing with the Mind’s Eye*. New York, NY: Random House.

[B77] Sheikh A. A. (1984). *Imagination and Healing.* New York, NY: Baywood.

[B78] Shiba Y. Santangelo A. M. Roberts A. C. (2016). Beyond the medial regions of prefrontal cortex in the regulation of fear and anxiety. *Front. Syst. Neurosci.* 10:12 10.3389/fnsys.2016.00012 PMC4761915 26941618

[B79] Shorr J. (1983). *Psychotherapy through Imagery.* New York, NY: Thieme-Stratton.

[B80] Simeone-DiFrancesco C. Roediger E. Stevens B. A. (2015). *Schema Therapy with Couples: A Practitioner’s Guide to Healing Relationships.* Chichester: John Wiley & Sons, 10.1002/9781118972700.ch10

[B81] Singer J. L. (1974). Daydreaming and the stream of thought. *Am. Sci.* 62 417–425. 4851267

[B82] Singer J. L. Pope K. S. (1978). “The use of imagery and fantasy techniques in psychotherapy,” in *The Power of Human Imagination*, eds Singer J. L. Pope K. S. (New York, NY: Plenum Press), 3–34. 10.1007/978-1-4613-3941-0

[B83] Singer J. L. Pope K. S. (1980). “The waking stream of consciousness,” in *The Psychobiology of Consciousness*, eds Davidson J. M. Davidson R. J. (New York: Plenum Press), 169–191. 10.1007/978-1-4684-3456-9-8

[B84] Steele K. Dorahy M. J. Van der Hart O. Nijenhuis E. R. (2010). “Dissociation versus alterations in consciousness: Related but different concepts,” in *Dissociation and the Dissociative Disorders: DSM-V and Beyond*, eds Dell P. F. O’Neil J. A. (New York, NY: Routledge), 155–170.

[B85] Stopa L. (2011). Imagery rescripting across disorders: a practical guide. *Cogn. Behav. Pract.* 18 421–423. 10.1016/j.cbpra.2011.05.001

[B86] Teasdale J. D. (1993). Emotion and two kinds of meaning: cognitive therapy and applied cognitive science. *Behav. Res. Ther.* 31 339–354. 10.1016/0005-7967(93)90092-9 8512536

[B87] Van Asselt A. D. Dirksen C. D. Arntz A. Severens J. L. (2007). The cost of border-line personality disorder: societal cost of illness in BPD-patients. *Eur. Psychiatry* 22: 354361. 10.1016/j.eurpsy.2007.04.001 17544636

[B88] Van der Hart O. Nijenhuis E. R. Steele K. (2006). *The Haunted Self.* New York, NY: WW Northon & Company.

[B89] Vonk R. (1999). Impression formation and impression management: motives, traits, and likeability inferred from self-promoting and self-deprecating behavior. *Soc. Cogn.* 17 390–412. 10.1521/soco.1999.17.4.390

[B90] Waltz P. G. Rapee R. M. (2003). Disentangling schematic and conceptual processing: a test of the interacting cognitive subsystems framework. *Cogn. Emot.* 17 65–81. 10.1080/02699930302273 29715742

[B91] Williams J. M. Watts F. N. MacLeod C. Oxford M. A. (1997). *Cognitive Psychology and Emotional Disorder.* Chichester: John Wiley & Sons.

[B92] Young J. E. (2002). “Schema-focused therapy for borderline personality disorder,” in *Cognitive Behavior Therapy: A Guide for the Practicing Clinician*, ed. Simos G. (New York, NY: Taylor & Francis Group), 201–227.

[B93] Young J. E. (2005). Schema-focused cognitive therapy and the case of Ms. S. *J. Psychother. Integr.* 15 115–126. 10.1037/1053-0479.15.1.115

[B94] Young J. E. Arnzt A. Atkinson T. Lobbestael J. Weishaar M. E. van Vreeswijk M. F. (2007). *The Schema Mode Inventory.* New York, NY: Schema Therapy Institute.

[B95] Young J. E. Klosko J. S. Weishaar M. E. (2003). *Schema Therapy: A Practitioner’s Guide.* New York, NY: Guilford Press, 10.1017/s1352465804211869

[B96] Zajonc R. B. (1980). Feeling and thinking preference need no inferences. *Am. Psychol.* 35 151–175. 10.1037/0003-066X.35.2.151

[B97] Zajonc R. B. (1984). On the primacy of affect. *Am. Psychol.* 39 117–123. 10.1037/0003-066X.39.2.117

